# The Prognostic Value of Positron Emission Tomography/Computed Tomography in Clinical Stage I Lung Cancer Patients: A Propensity-Match Analysis

**DOI:** 10.3390/jcm13082416

**Published:** 2024-04-21

**Authors:** Ya-Fu Cheng, Jing-Yang Huang, Ching-Hsiung Lin, Sheng-Hao Lin, Bing-Yen Wang

**Affiliations:** 1 Division of Thoracic Surgery, Department of Surgery, Changhua Christian Hospital, Changhua 500, Taiwan; 181033@cch.org.tw; 2 Institute of Medicine, Chung Shan Medical University, Taichung 402, Taiwan; 3 Division of Chest Medicine, Department of Internal Medicine, Changhua Christian Hospital, Changhua 500, Taiwan; 47822@cch.org.tw (C.-H.L.);

**Keywords:** positron emission tomography/computed tomography, prognosis, lung cancer

## Abstract

**Background:** The application of positron emission tomography/computed tomography (PET/CT) helps provide accurate clinical staging for lung cancer patients. However, the effects and trends in early-stage lung cancer remain unclear. The aim of this study was to compare differences between clinical stage I lung cancer patients who received PET/CT for staging and those who did not. **Methods:** Data were obtained from the Taiwan Society of Cancer Registry. There were 6587 clinical stage I lung cancer patients between 2009 and 2014 analyzed in this study. We compared the characteristics of the PET/CT and no PET/CT groups. After propensity score matching, it resulted in both groups having 2649 patients. We measured the overall survival rates of all clinical stage I lung cancer patients and the overall survival rates of patients with PET/CT and without PET/CT. **Results:** The 1-, 3-, and 5-year survival rates of all clinical stage I lung cancer patients were 97.2%, 88.2%, and 79.0%, respectively. Patients with a larger tumor size tended to receive PET/CT for staging (stage Ib: 38.25% vs. 27.82%, *p* < 0.0001) and a larger resection (lobectomy: 74.62% vs. 66.61%, *p* < 0.0001). The 5-year survival rates were 79.8% in the PET/CT group and 78.2% in the no PET/CT group after propensity score matching (*p* = 0.6528). **Conclusions:** For clinical stage I lung cancer in Taiwan, patients with larger tumor sizes tend to have PET/CT for staging. Although PET/CT provided more precise clinical staging, these patients still received larger resections and had more pathological migration. However, there was no overall survival rate benefit after PET/CT.

## 1. Introduction

Lung cancer is identified as the leading cause of cancer death worldwide [1]. Around three-quarters of lung cancer cases are diagnosed at a late stage, and less than 20% of lung cancer cases are diagnosed at stage I [2]. Through the popularity of lung cancer screenings nowadays, smaller nodules are being detected, and the algorithms for early-stage lung cancer have grown more complex. Both planning and prognosis are dependent on precise staging. The pretreatment evaluation of stage I lung cancer includes pulmonary function tests, a bronchoscopy, mediastinal lymph node evaluation, brain magnetic resonance imaging (MRI) with contrast, and positron emission tomography/computed tomography (PET/CT) [3].

Computed tomography (CT) with contrast can help in the early detection of nodules and the evaluation of lymphadenopathy, the solid component ratio, and density. However, it is of limited use in differentiating between benign and malignant lesions [4]. The combination of PET and CT scans was introduced into clinical practice in 1998 [5]. Previous studies have shown that it cannot provide better sensitivity (89% vs. 94%) or specificity (78% vs. 73%) compared to CT for differentiating between benign and malignant solitary pulmonary nodules [6]. However, the application of PET/CT directly improved the clinical staging in 25% of non-small-cell lung cancer patients and 29% of small-cell lung cancer patients and often led to a change in the treatment choice [7,8]. Furthermore, PET/CT helps to differentiate between benign and malignant pulmonary nodules [9]. Though there are so many benefits after PET/CT evaluation, the effects and trends in early-stage lung cancer remain unclear.

For this study, we obtained data from the Taiwan Society of Cancer Registry (TSCR) over a 5-year period. We aimed to compare the differences between clinical stage I lung cancer patients who received PET/CT for staging and those who did not.

## 2. Materials and Methods

### 2.1. Study Design

The study was approved by the Institutional Review Board in our institution (IRB-161222, approval date: 23 March 2017), and informed consent from all participants was waived. The population data were obtained from the TSCR. These data include the entire population of 23 million people in Taiwan, with registration files and original claims data for each patient. All the patients were strictly confirmed by tissue diagnosis. We searched data between January 2009 and December 2014. The end date of survival follow-up was December 2017. The median follow-up time was 64.3 months in the PET/CT group and 65.6 months in the no PET/CT group.

### 2.2. Patients

**Figure 1:**
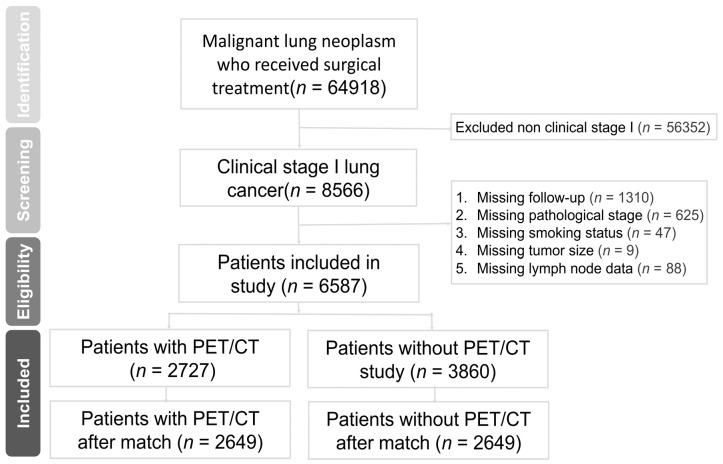
Flow chart of patient recruitment in the study.

We identified patients who were diagnosed with lung cancer by the diagnostic codes C34.0, C34.1, C34.2, C34.3, C34.8, and C34.9. A total of 64,918 patients with malignant lung neoplasms who received surgical treatment were identified (Figure 1). There were 8566 patients who were diagnosed at clinical stage I. A total of 1979 patients were excluded from the study. Among these, 1310 patients had a missing follow-up 3 months post-operation, 625 had a missing pathological stage, 47 patients had a missing smoking status, 9 patients had a missing tumor size, and 88 patients had missing lymph node data. Therefore, a total of 6587 patients were enrolled into the study. There were 2727 patients who received PET/CT, and the other 3860 patients did not receive PET/CT.

The following items were included in the study: age, sex, smoking status, cell type, operative method, clinical stage, pathological stage, and treatment. The primary objective was to find different features and characteristics of the two groups. The secondary objective was to compare the overall survival rates between the two groups.

In the great majority of surgical practice in our country, surgeons tend to carry out both N1 lymph node dissections and three stationary mediastinal lymph node dissections (N2) for clinical stage I NSCLC.

### 2.3. Statistical Analysis

We used SAS software (SAS System for Windows, version 9.2; SAS Institute, Cary, NC, USA) to perform the statistical analysis for this study. The PET/CT and no PET/CT groups were compared using Wilcoxon rank-sum tests for continuous variables and chi-squared or Fisher’s exact tests for categorical variables. In order to reduce the bias, we used propensity score matching of age, sex, smoking status, cell type, operative method, clinical stage, and pathological stage. There were 2649 patients in both groups after the propensity match.

Survival curves were plotted using the Kaplan–Meier method, and the difference in survival was calculated by the log-rank test. The survival rates of pathological stages I, II, and III and the 1-, 3-, and 5-year survival rates of all clinical stage I lung cancer patients were analyzed.

Univariate and multivariate analyses were performed with the Cox proportional hazards model. Covariates were selected based on clinical judgment. The following factors were included in the analyses: age, sex, smoking status, cell type, operative method, clinical stage, pathological stage, and PET/CT performance. Statistical analysis with a *p*-value less than 0.05 was considered statistically significant.

## 3. Results

Data from January 2009 to December 2014 for 6587 clinical stage I lung cancer patients were analyzed. Among these, 2727 patients received PET/CT, and the other 3860 patients did not receive PET/CT. After propensity score matching, there were 2649 patients in both groups (Table 1).

The mean age of the PET/CT group was older (63.63 years old) than that of the no-PET/CT group (62.10 years old, *p* < 0.0001). Both groups were female-predominant (54.75% in the PET/CT group and 56.32% in the no-PET/CT group).

The predominant cell type of the clinical stage I lung cancer patients was adenocarcinoma (AD) (PET/CT group, n = 2191, 80.34%; no PET/CT group, n = 3027, 78.42%, *p* < 0.0001). The patients in the PET/CT group underwent a lobectomy at a significantly higher rate than the patients in the no-PET/CT group (n = 2035, 74.62% vs. n = 2571, 66.61%, *p* < 0.0001). Only 307 patients (11.26%) in the PET/CT group underwent wedge resection compared to 778 patients (20.16%) in the no-PET/CT group. In regard to pathological stage, there were only 2229 patients (81.74%) remaining at pathological stage I in the PET/CT group and 3407 such patients (88.26%) in the no-PET/CT group (*p* < 0.0001).

**Figure 2:**
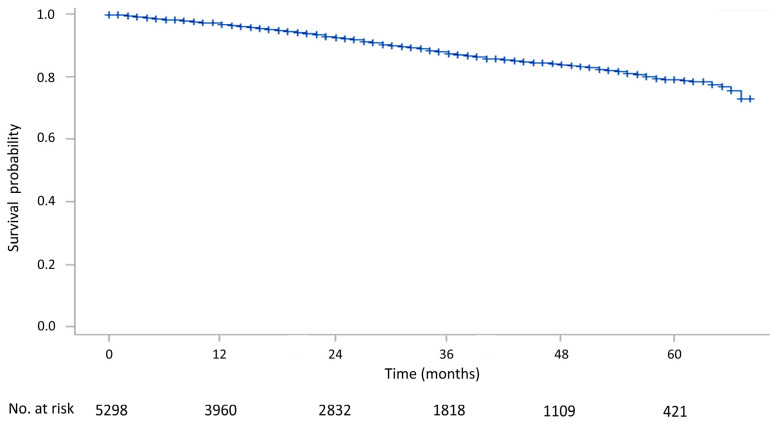
The overall survival rates of clinical stage I lung cancer patients.

**Figure 3:**
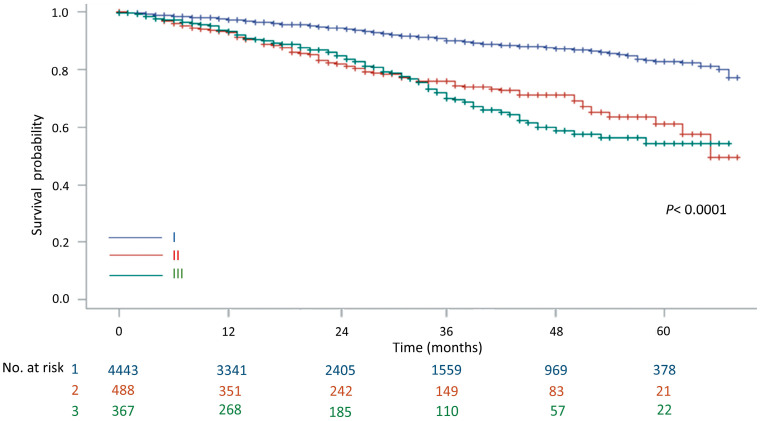
The overall survival rates of pathological stage I, II, and III lung cancer patients who were diagnosed as clinical stage I and received surgery.

The 1-, 3-, and 5-year survival rates of all the clinical stage I lung cancer patients were 97.2%, 88.2%, and 79.0%, respectively (Figure 2). The 5-year survival rates were significantly different between pathological stages I, II, and III among those who were diagnosed as clinical stage I and received surgery (*p* < 0.0001). The 5-year survival rate was 83.8% in pathological stage I, 62.5% in pathological stage II, and 53.6% in pathological stage III (Figure 3).

**Figure 4:**
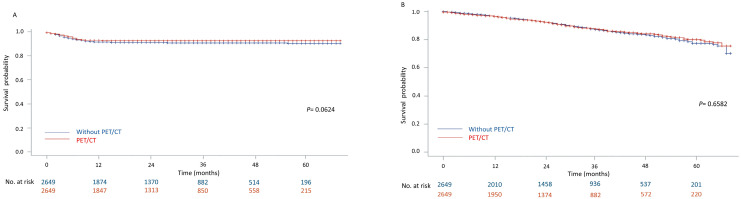
The (**A**) time-to-recurrence and (**B**) overall survival rates of clinical stage I lung cancer patients with PET/CT staging and without PET/CT staging after propensity score matching.

After the propensity score matching, we analyzed the outcomes between the PET/CT group and the no-PET/CT group. Most recurrence occurred in the first year (7.24% in the PET/CT group vs. 8.27% in the no-PET/CT group) (Figure 4A). The 1-, 3-, and 5-year survival rates were not significantly different when comparing the PET/CT group and the no PET/CT group (*p* = 0.6528). The 1-, 3-, and 5-year survival rates were 97.0%, 88.2%, and 79.8% in the PET/CT group and 97.5%, 88.1%, and 78.2% in the no-PET/CT group (Figure 4B).

We used univariate and multivariate analyses to find independent factors to predict the clinical stage I lung cancer survival rate (Table 2 and Table 3). Older age (>60 years old), male sex, being currently a smoker, SqCC cell type, clinical stage Ib, and advanced pathological stage were independent factors of a poor 5-year survival rate. Patients between 60 and 69 years of age (univariate: HR = 1.936; multivariate: HR = 1.511) and patients over 70 years old (univariate: HR = 3.715; multivariate: HR = 2.195) had worse 5-year survival rates than those younger than 50 years of age in both the univariate and multivariate analyses.

Males had a lower 5-year survival rate than females (univariate: HR = 2.341; multivariate: HR = 1.436). Patients who never smoked had a better 5-year survival rate than those who quit smoking (univariate: HR = 1.959; multivariate: HR = 1.357) and current smokers (univariate: HR = 3.045; multivariate: HR = 1.600).

Most importantly, the patients who received PET/CT did not have a significantly different 5-year survival rate compared to the patients who did not receive PET/CT (univariate: *p* = 0.1023; multivariate: *p* = 0.9105).

## 4. Discussion

Our study was a retrospective study investigating the prognosis of clinical stage I lung cancer patients in Taiwan after receiving PET/CT. Previous studies have shown lots of prognostic factors in lung cancer, such as age, sex, stage, performance status, tumor differentiation, and lactate dehydrogenase [10,11]. Our study revealed that older age, male sex, being currently a smoker, SqCC cell type, clinical stage Ib, and advanced pathological stage were independent factors of a poor overall survival rate. However, PET/CT was not an independent factor.

Focusing on PET/CT, it is widely used in the evaluation of stage I lung cancer. A previous study demonstrated that PET/CT improves the diagnostic accuracy of the staging of non-small-cell lung cancer compared to CT alone [12]. It can provide more accurate tumor staging, nodal staging, and metastasis status. The sensitivity and specificity for evaluating N staging are 73% and 91% with PET/CT, compared to 74% and 73% for CT alone [13]. With more accurate clinical staging, it was believed that pathological stage migration would be lessened in the PET/CT group. However, we observed the opposite results. We believe this is due to selection bias by the doctors. The tumor size in the PET/CT group was much larger than in the no-PET/CT group in this study. This means that the doctors tended to arrange PET/CT staging prior to surgery when the tumor was larger. We also noted that the patients in the PET/CT group tended to receive a lobectomy more. With a larger resection and more lymph nodes retrieved, stage migration is more likely to occur. There are several factors that might cause clinical and pathological stage migration in lung cancer, including time from diagnosis to surgery, clinical T stage, and the number of lymph nodes obtained [14,15]. We suggest that the effects of a larger tumor size and more lobectomies prevailed over the effect of PET/CT evaluation. When there is a larger tumor size or a suspicion of a malignant tumor, lobectomy and thorough lymph node dissection should be carried out even if PET/CT revealed no stage migration.

Although much pathological stage migration is still noted, there are still several advantages after the application of PET/CT. First, it can lead to the clinical stage migration of a lung cancer patient before surgery. Gregory et al. analyzed 168 non-small-cell lung cancer (NSCLC) patients and showed that there were clinical stage migrations in 50.6% (41.1% upstaged, 9.5% downstaged) of them after the application of PET/CT [16]. Clinical stage migrations occurred for 12% to 44% of small-cell lung cancer (SCLC) patients after receiving PET/CT [17,18]. These significant clinical stage migrations might reduce the number of pathological stage migrations. Furthermore, most restaging after receiving PET/CT resulted in management and prognosis alterations.

Second, the use of PET/CT reduces futile treatments and their associated morbidity, thus reducing costs. Several previous studies claimed that there were economic benefits to PET/CT in the management of patients with lung cancer [19,20,21,22]. Schreyogg et al. concluded that the incremental cost-effectiveness ratio (ICER) was 3508 USD per NSCLC patient when comparing PET/CT to CT alone [21]. Similarly, another randomized clinical trial of 189 NSCLC patients showed that the ICER was estimated at 3927 EUR when patients received PET/CT [22].

Lastly, the management strategies were also changed after PET/CT due to stage migration. Taus et al. reported that among NSCLC patients who received PET/CT, 34.6% had stage migration, 24.4% had treatment modifications, and 5.2% avoided futile thoracotomies [23]. Kubota et al. even showed that PET/CT contributed to modifications in management strategies in 71.6% of lung cancer patients [24]. Our data indicated that the clinical stage I lung cancer patients who received PET/CT had a higher lobectomy rate and a lower wedge resection rate. Moreover, patients were more likely to receive chemotherapy after receiving PET/CT. The tumor size was larger in the PET/CT group, and pathological stage migrations were more frequent; we assumed these were the reasons there were more lobectomies and adjuvant chemotherapy in the PET/CT group.

While PET/CT was introduced for pretreatment evaluation, a high proportion of the patients receiving PET/CT had pathological stage migration. Theoretically, PET/CT bringing more accurate clinical staging might result in less pathological stage migration and a better prognosis for these patients. However, our data showed that there was more pathological stage migration and a similar 5-year survival rate. There are few studies reporting the effects of PET/CT on the prognosis of lung cancer patients. In the real world, PET/CT was arranged when the tumor was more likely to be larger or advanced. In these cases, pathological stage migration was more likely to occur even after PET/CT provided more precise clinical staging. A more accurate evaluation, like endobronchial ultrasound-guided transbronchial-fine needle aspiration (EBUS/TBNA) or using a mediastinal scope during a lymph node biopsy, might be necessary in these patients.

There are some limitations to our study. First, this is a retrospective study. Although there was a huge amount of data, a prospective study is more convincing. Second, there might be a selection bias due to the varying usage of PET/CT scans among doctors in Taiwan. Some doctors preferred to perform PET/CT scans before the surgery, and others did not. Third, there are so many factors that influence the results of lung cancer survival rates, including driver mutations, tumor subtype, lymph and vascular invasion, tumor doubling time, and spread through air spaces (STAS). They are hard to describe and discuss accurately. Fourth, our national health insurance only covers the cost of PET/CT staging for lung cancer patients who might have a change in treatment strategy. Although we suggest most clinical stage I lung cancer patients would still be covered, it might affect a surgeon’s decision to perform PET/CT for staging. Last but not least, we could not obtain the time-to-surgery data in the TSCR database. It usually takes about 2 to 4 weeks for PET/CT scan results to be finalized in our country. This prolonged evaluation time might be related to upstaging.

## 5. Conclusions

For clinical stage I lung cancer in Taiwan, patients with larger tumor sizes tend to have PET/CT for staging. Although PET/CT provided more precise clinical staging, these patients still received larger resections and had more pathological migration. However, there is no overall survival rate benefit after PET/CT.

## Figures and Tables

**Table 1:** Demographic data of patients with clinical stage I lung cancer.

All Patients				Propensity-Matched Patients		
Characteristics	with PET/CT	without	*p*	with PET/CT	without	*p*
Number	2727	3860		2649	2649	
Age (years)	63.63 ± 11.12	62.10 ± 11.08	<0.0001	63.55 ± 11.12	63.24 ± 11.04	0.9668
<50	302 (11.07%)	485 (12.56%)		291 (10.99%)	302 (11.40%)	
50–59	655 (24.02%)	1083 (28.06%)		647 (24.42%)	647 (24.42%)	
60–69	885 (32.45%)	1231 (31.89%)		862 (32.54%)	852 (32.16%)	
>=70	885 (32.45%)	1061 (27.49%)		849 (32.05%)	848 (32.01%)	
Sex			0.2058			0.3929
Male	1234 (45.25%)	1686 (43.68%)		1206 (45.53%)	1237 (46.70%)	
Female	1493 (54.75%)	2174 (56.32%)		1443 (54.47%)	1412 (53.30%)	
Smoking status			<0.0001			0.6295
Never	1303 (47.78%)	2113 (54.74%)		1274 (48.09%)	1246 (47.04%)	
Current	255 (9.35%)	350 (9.07%)		249 (9.40%)	266 (10.04%)	
Quit	1169 (42.87%)	515 (13.34%)		1126 (42.51%)	1137 (42.92%)	
Cell type			<0.0001			0.9990
SqCC	263 (9.64%)	318 (8.24%)		258 (9.74%)	258 (9.74%)	
AD	2191 (80.34%)	3027 (78.42%)		2120 (80.03%)	2121 (80.07%)	
Others	273 (10.01%)	515 (13.34%)		271 (10.23%)	270 (10.19%)	
Treatment			<0.0001			0.7002
Lobectomy	2035 (74.62%)	2571 (66.61%)		1974 (74.52%)	1971 (74.41%)	
Wedge	307 (11.26%)	778 (20.16%)		306 (11.55%)	292 (11.02%)	
Others	385 (14.12%)	511 (13.24%)		369 (13.93%)	386 (14.57%)	
Clinical stage			<0.0001			0.3924
Ia	1684 (61.75%)	2786 (72.18%)		1663 (62.78%)	1693 (63.91%)	
Ib	1043 (38.25%)	1074 (27.82%)		986 (37.22%)	956 (36.09%)	
Pathological stage			<0.0001			0.3462
I	2229 (81.74%)	3407 (88.26%)		2202 (83.13%)	2241 (84.60%)	
II	285 (10.45%)	259 (6.71%)		255 (9.63%)	233 (8.80%)	
III	213 (7.81%)	194 (5.03%)		192 (7.25%)	175 (6.61%)	
Survival rate						
1 year	0.9691	0.9789	0.0703	0.9699	0.9745	0.7243
3 years	0.8778	0.8951	0.0460	0.8819	0.8814	0.9145
5 years	0.7921	0.8020	0.1247	0.7976	0.7822	0.6528

SqCC: squamous cell carcinoma; AD: adenocarcinoma.

**Table 2:** Univariate analysis of overall survival rates.

Variables	HR	95% Confidence Interval	*p* Value
Age			
<50	reference		
50–59	1.115	(0.757–1.641)	0.5815
60–69	1.936	(1.353–2.769)	0.0003
≥70	3.715	(2.629–5.247)	<0.0001
Sex			
Female	reference		
Male	2.341	(1.983–2.763)	<0.0001
Smoking status			
Never	reference		
Current	3.045	(2.253–4.114)	<0.0001
Quit	1.959	(1.585–2.422)	<0.0001
Cell type			
Adenocarcinoma	reference		
SqCC	3.217	(2.645–3.911)	<0.0001
Others	1.702	(1.338–2.165)	<0.0001
Treatment			
Lobectomy		reference	
Wedge resection	1.497	(1.206–1.858)	0.0003
Others	1.192	(0.957–1.483)	0.1164
Clinical stage			
Ia	reference		
Ib	2.353	(2.006–2.759)	<0.0001
Pathological stage			
I	reference		
II	2.954	(2.390–3.650)	<0.0001
III	3.510	(2.812–4.381)	<0.0001
PET/CT			
No	reference		
Yes	1.142	(0.974–1.340)	0.1023

HR = hazard ratio.

**Table 3:** Multivariate analysis of 5-year survival rates.

Variables	HR	95% Confidence Interval	*p* Value
Age			
<50	reference		
50–59	1.004	(0.680–1.481)	0.9852
60–69	1.511	(1.053–2.168)	0.0252
≥70	2.195	(1.534–3.141)	<0.0001
Sex			
Female	reference		
Male	1.436	(1.187–1.737)	0.0002
Smoking status			
Never	reference		
Current	1.600	(1.158–2.210)	0.0044
Quit	1.357	(1.083–1.700)	0.0080
Cell type			
Adenocarcinoma	reference		
SqCC	1.381	(1.107–1.723)	0.0042
Others	1.238	(0.964–1.589)	0.0950
Treatment			
Lobectomy	reference		
Wedge resection	1.599	(1.275–2.004)	<0.0001
Others	1.080	(0.865–1.349)	0.4972
Clinical stage			
Ia	reference		
Ib	1.235	(1.009–1.512)	0.0410
Pathological stage			
I	reference		
II	1.900	(1.301–2.775)	0.0009
III	2.333	(1.424–3.821)	0.0008
PET/CT			
No	reference		
Yes	0.991	(0.844–1.164)	0.9105

HR = hazard ratio.

## Data Availability

The raw data supporting the conclusions of this article will be made available by the authors upon request.
